# A Protective Factor Against Mental Health Problems in Youths? A Critical Note on the Assessment of Self-Compassion

**DOI:** 10.1007/s10826-015-0315-3

**Published:** 2015-11-04

**Authors:** Peter Muris

**Affiliations:** Clinical Psychological Science, Faculty of Psychology and Neuroscience, Maastricht University, P.O. Box 616, 6200 MD Maastricht, The Netherlands; Department of Psychology, Stellenbosch University, Stellenbosch, South Africa

**Keywords:** Self-compassion, Psychopathology, Youths, Assessment

## Abstract

Self-compassion, which refers to the tendency of being kind and understanding to oneself when confronted with personal failings and difficulties, is increasingly investigated as a protective factor within the context of mental health problems. In this invited paper, I will briefly introduce the concept of self-compassion and give an overview of the research that has examined its relationship with psychopathology in youth. Then I will make my critical point regarding the assessment of self-compassion: the scales that are currently used for measuring this construct include a large number of reversely scored, negative items that measure the precise opposite of having compassion with oneself. I present evidence (partly on the basis of own data) that these negative items do not reflect the true protective nature of self-compassion and tend to inflate the relation with psychopathology. My recommendation is to remove the negative items from the scales and to assess self-compassion by means of a set of items that truly reflect its protective nature.

## Introduction

Researchers on child and adolescent psychopathology increasingly acknowledge the fact that not only risk and vulnerability factors are important for understanding the development of internalizing and externalizing problems in young people, but that protective factors are also highly relevant (e.g., Masten et al. 2009). Self-compassion, which can be briefly defined as being kind and understanding to oneself when confronted with personal failings and difficulties (Neff 2015), is one of the protective psychological variables that has received considerable empirical attention within the context of mental health problems in adults (Barnard and Curry 2011; MacBeth and Gumley 2012). During the past years, researchers have started to explore self-compassion as a correlate of psychological maladjustment in youth populations as well. In this invited paper, I will first discuss the concept of self-compassion in somewhat more detail. Next, I will give a short overview of the research that has been conducted on self-compassion and psychopathology in youths. Following this, a critical note will be made regarding the assessment of self-compassion, which is corroborated by means of own research data. Finally, the implications of this critique will be discussed and a recommendation for the measurement of self-compassion in future studies is made.

## Self-Compassion

Without any doubt it was Kristin Neff who gave the research on self-compassion an enormous boost. This American professor in psychology, currently working at the University of Texas at Austin, was the first to systematically investigate self-compassion in relation to people’s mental health problems. Neff produced a considerable number of scientific publications, while also advocating the protective power of this self-related concept in the popular media. According to Neff (2003), self-compassion consists of three key components. The first component is self-kindness and refers to the tendency to be caring to and understanding with oneself when confronted with personal failures. The second component is common humanity, which is concerned with the inclination to recognize that one’s failures and problems are a normal part of human life. The third and final component is mindfulness and has to do with the ability of not becoming too absorbed with personal failures and problems but rather to maintain a balanced view on what goes right and what goes wrong.

Since its introduction, more than a decade ago, self-compassion has been increasingly examined as a protective factor within the context of mental health in adults. Most of the evidence obtained in this research indicates that this construct is indeed inversely related to psychological well-being: individuals with higher levels of self-compassion generally exhibit lower levels of anxiety, depression, stress, and other psychopathological symptoms (e.g., MacBeth and Gumley 2012). Of particular interest is that self-compassion may also provide a lead for clinical interventions: by promoting these self-soothing skills, psychological health can be enhanced. This idea has not only been embraced by Acceptance and Commitment Therapy which currently includes self-compassion as one of its main targets (Hayes et al. 2012), but there are also treatment programs that specifically focus on the enhancement of this self-related construct (e.g., Gilbert 2009; Neff and Gerner 2013; Smeets et al. 2014).

## Self-Compassion and Psychopathology in Youths

A systematic search carried out in the Web-of-Science database with [SELF-COMPASSION] and [CHILD* or ADO*] as search terms learns that so far nine studies have investigated self-compassion in relation to mental health problems in young people. As can be seen in Table 1, studies were conducted with a total of 3477 young participants (1864 boys and 1613 girls) who were predominantly of an adolescent age (range between 11 and 20 years). Five of the studies were conducted with randomly recruited middle and high school students. The other four investigations made use of at-risk samples of youths who (a) had a history of maltreatment (Tanaka et al. 2011), (b) poorly performed at school and thus were prone to victimization (Jativa and Cerezo 2014), (c) attended a residential program (Barry et al. 2015), or (d) had been exposed to a potentially traumatic event (a forest fire disaster; Zeller et al. 2015). In terms of type of psychopathology, all studies incorporated measures for assessing internalizing symptoms such as anxiety, depression, stress etc., but a number of investigations also included measures of externalizing problems such as aggression and substance use.

**Table 1:** Overview of the studies that have examined the relation between self-compassion and symptoms of psychopathology in youths

Study	Sample characteristics	*N* (boys/girls)	*M* age (range)	Assessment of self-compassion	Psychopathology	*r* with self-compassion
Neff and McGehee (2010)	High school students in the United States	235 (113/122)	15.2 years (14–17)	SCS	Anxiety	−0.60
					Depression	−0.73
Tanaka et al. (2011)	Maltreated youths receiving child protective services in Canada	117 (53/64)	18.1 years (16–20)	SCS	Alcohol use	−0.21
					Depression	−0.37
					Psychological distress	−0.33
					Substance use	−0.11
					Suicide attempt	−0.30
Bluth and Blanton (2014a)	High school students in the United States	67 (28/39)	NR (14–18)	SCS	Negative affect	−0.64
					Stress	−0.70
Bluth and Blanton (2014b)	Middle and high school students in the United States	90 (40/50)	NR (11–18)	SCS	Negative affect	−0.60
					Stress	−0.70
Jativa and Cerezo (2014)	Poor performing high school students in Spain	109 (78/31)	16.7 years (15–18)	SCS	Externalizing problems	−0.24
					Internalizing problems	−0.52
Barry et al. (2015)	Adolescents attending a voluntary residential program in the United States	215 (215/0)	16.8 years (16–18)	SCS	Aggression	−0.22
					Anxiety	−0.32
					Depression	−0.27
					Narcissism	−0.32
Marshall et al. (2015)	High school students in Australia	2448 (1234/1214)	14.7 years (NR)	SCS-SF	Mental health problems	−0.39
Zeller et al. (2015)	At-risk sample of adolescents exposed to a potentially traumatic event in Israel	64 (47/17)	17.5 years (15–19)	SCS	Depression	−0.39*
					Panic	−0.38*
					Posttraumatic stress	−0.25*
					Suicidality	−0.29*
Muris et al. (in press)	High school students in the Netherlands	132 (56/76)	14.8 years (12–17)	S-SCS-A (only positive components)	Anxiety	−0.26
					Depression	−0.35

*NR* not reported, *SCS* Self-Compassion Scale, *SCS-SF* Self-Compassion Scale-Short Form, *S-SCS-A* Shortened Self-Compassion Scale for Adolescents

* These correlations were not reported in the paper by Zeller et al. (2015) but estimated on the output of multi-level analyses. As these analyses also controlled for other variables, the actual correlations between self-compassion and these psychopathology indices are likely to be higher

The correlations between self-compassion and various symptoms of psychopathology, as documented in these studies, are shown in the right column of Table 1. As can be seen, correlations were all negative, thus indicating that higher levels of self-compassion were associated with lower levels of psychopathology and vice versa. Using Wilson’s (2010) online meta-analysis calculator, correlations were transformed into effect sizes (*r*), which were averaged for each study and then pooled across all studies. The overall effect size for the relationship between self-compassion and symptoms of psychopathology in young people was large, *r* = −0.49 (95 % CI −0.67 to −0.32), and comparable to the pooled effect size of *r* = −0.54 as reported by MacBeth and Gumley (2012) in their meta-analysis of studies conducted with adults. Thus, the results of research carried out in young people correspond nicely with what has been reported in the adult literature, and seem to be in keeping with the notion that self-compassion is a protective factor against psychopathology.

## A Critical Note on the Assessment of Self-Compassion

Researchers widely rely on Neff’s (2003) Self-Compassion Scale (SCS) or its abbreviated version, the Self-Compassion Scale-Short Form (SCS-SF; Raes et al. 2011), for measuring this construct. Critique can be raised regarding the validity of these scales because half of the items do not measure the three key components of self-kindness, common humanity, and mindfulness, but rather assess their precise counterparts of self-judgment, isolation, and over-identification. These negative items, which are reversed before adding them to the positive items to produce a total self-compassion score, are problematic as they seem to measure characteristics that are already known to be associated with psychopathology. More specifically, self-judgment shows clear similarity with harsh self-criticism (e.g., Zuroff et al. 1990), isolation shares features with social withdrawal and loneliness (e.g., Rubin and Coplan 2004), whereas over-identification matches with self-absorption and self-focused rumination (e.g., Lyubomirsky and Nolen-Hoeksema 1995), all of which have been demonstrated to be pervasive features of mental health problems, especially those of an internalizing nature. Thus, it can be argued that the widely used SCS and its abbreviated version, the SCS-SF, may not be optimal instruments for measuring the true protective nature of self-compassion, mainly because these scales include negative items that tap toxic mechanisms which may inflate the relationship with psychopathology.

In general, there seems to be little awareness among researchers of this assessment problem with the SCS or SCS-SF. A recent review of the literature (Muris and Petrocchi submitted) indicated that the vast majority of the studies (83.5 %) solely relied on the SCS or SCS-SF total score as an index of self-compassion. A meta-analysis of the investigations that did have an eye for the various components within these scales confirmed that the inversely scored negative components of self-judgment, isolation, and over-identification were more convincingly related to psychopathology than the positive components of self-kindness, common humanity, and mindfulness (with effect size *r*’s ranging between −0.47 and −0.50 vs. −0.27 and −0.34). This demonstrates that although the protective influence of ‘true’ self-compassion certainly seems to exist, this effect is likely to be boosted when the assessment also includes the aforementioned negative components.

So far, studies on the relation between self-compassion and psychopathology in youths have also largely neglected this assessment issue with the SCS or SCS-SF. Of the eight studies that used the original SCS or SCS-SF (see Table 1), only Barry et al. (2015) reported on the links between separate self-compassion components and psychopathology in their sample of residential adolescent males. Their data confirm that the negative components were more substantially associated with psychopathology indices than the positive components, but the researchers remain silent about the implications of this result. In a recently conducted study, I was able to directly examine the differential associations between the negative and positive components of self-compassion and psychopathology in young people. One-hundred-and-eighty-four high school students aged 12–16 years completed the SCS-SF and Achenbach’s (2009) Youth Self-Report for measuring internalizing and externalizing symptoms. As displayed in Table 2, total self-compassion showed the expected negative correlations with psychopathology, although the link with internalizing was clearly more substantial than that with externalizing (*Z* = 4.10, *p* < 0.001). Most importantly, in the case of internalizing, the negative components of self-compassion were stronger correlates of symptoms than the positive components (*Z* = 4.69, *p* < 0.001). An additional analysis revealed that a total of 38.2 % of the variance in internalizing symptoms could be explained by self-compassion. However, the contribution of the positive components was only 6.1 %, whereas the remaining 32.1 % was attributable to the reversely scored negative components. In the case of externalizing, the positive and negative components appeared to be equally strong correlates of symptoms, and here the inclusion of the negative components had less influence on the total variance explained (i.e., 2.2 of 6.5 %). In conclusion, these data are the first to actually demonstrate that the negative components in measures such as the SCS and SCS-SF produce inflated correlations with psychopathology. This inflation effect seems to be stronger for internalizing than for externalizing symptoms, but obviously this impression needs further investigation.

**Table 2:** Results of an unpublished study on the relationship between various indices of self-compassion as measured with SCS-SF and internalizing (anxiety, depression, and somatization) and externalizing (oppositional-defiant disorder and conduct disorder) symptoms of non-clinical adolescents (*N* = 184)

Total self-compassion	Positive components			Negative components^a^		
	Self-kindness	Common humanity	Mindfulness	Self-judgment	Isolation	Over-identification
*Internalizing symptoms*						
−0.56***	−0.25**			−0.61***		
	−0.23**	−0.19**	−0.20**	−0.51***	−0.59***	−0.45***
*Externalizing symptoms*						
−0.25**	−0.21**			−0.19**		
	−0.16*	−0.08	−0.28***	−0.19**	−0.20**	−0.09

*SCS-SF* Self-Compassion Scale-Short Form

^a^Reversely scored

* *p* < 0.05; ** *p* < 0.01; *** *p* < 0.001

## Conclusion

It is not my intention to disqualify self-compassion as a protective mechanism within the context of mental health problems and I do hope that researchers will continue to explore its relevance for youth psychopathology. I only want to raise a critical point regarding the assessment of this construct: by including negative components such as self-judgment, isolation, and over-identification, scales like the SCS and SCS-SF tap a number of toxic mechanisms that do not fit with the true, protective nature of self-compassion and inflate its link with psychopathology. When we recently submitted work to the *Journal of Child and Family Studies* in which we argued to only use the positive components of the SCS and to discard the negative components (Muris et al. in press), two of the three reviewers were rather skeptical about this idea, with one of them firmly stating that “the construct of self-compassion entails 6 dimensions” and that “the 3 “negative” dimensions should not be removed”. I hope that this paper convinces these reviewers as well as other researchers, and that they will follow my recommendation to rely on instruments that merely assess the positive components of self-compassion as this seems to be the best way of capturing the pure, protective qualities of this interesting self-related construct.
